# Pharmacological Inhibition of IRE-1 Alpha Activity in Herpes Simplex Virus Type 1 and Type 2-Infected Dendritic Cells Enhances T Cell Activation

**DOI:** 10.3389/fimmu.2021.764861

**Published:** 2022-01-05

**Authors:** Eduardo I. Tognarelli, Angello Retamal-Díaz, Mónica A. Farías, Luisa F. Duarte, Tomás F. Palomino, Francisco J. Ibañez, Claudia A. Riedel, Alexis M. Kalergis, Susan M. Bueno, Pablo A. González

**Affiliations:** ^1^ Millennium Institute on Immunology and Immunotherapy, Departamento de Genética Molecular y Microbiología, Facultad de Ciencias Biológicas, Pontificia Universidad Católica de Chile, Santiago, Chile; ^2^ Millennium Institute on Immunology and Immunotherapy, Departamento de Biotecnología, Facultad de Ciencias del Mar y de Recursos Biológicos, Universidad de Antofagasta, Antofagasta, Chile; ^3^ Millennium Institute on Immunology and Immunotherapy, Departamento de Biología Celular, Facultad de Ciencias de la Vida, Universidad Andrés Bello, Santiago, Chile; ^4^ Millennium Institute on Immunology and Immunotherapy, Departamento de Endocrinología, Facultad de Medicina, Escuela de Medicina, Pontificia Universidad Católica de Chile, Santiago, Chile

**Keywords:** dendritic cells, HSV-1, HSV-2, unfolded protein response (UPR), IRE-1α, T-cell activation, adaptive immunity, apoptosis

## Abstract

Herpes simplex virus type 1 (HSV-1) and type 2 (HSV-2) infections are life-long and highly prevalent in the human population. These viruses persist in the host, eliciting either symptomatic or asymptomatic infections that may occur sporadically or in a recurrent manner through viral reactivations. Clinical manifestations due to symptomatic infection may be mild such as orofacial lesions, but may also translate into more severe diseases, such as ocular infections that may lead to blindness and life-threatening encephalitis. A key feature of herpes simplex viruses (HSVs) is that they have evolved molecular determinants that hamper numerous components of the host’s antiviral innate and adaptive immune system. Importantly, HSVs infect and negatively modulate the function of dendritic cells (DCs), by inhibiting their T cell-activating capacity and eliciting their apoptosis after infection. Previously, we reported that HSV-2 activates the splicing of the mRNA of XBP1, which is related to the activity of the unfolded protein response (UPR) factor Inositol-Requiring Enzyme 1 alpha (IRE-1α). Here, we sought to evaluate if the activation of the IRE-1α pathway in DCs upon HSV infection may be related to impaired DC function after infection with HSV-1 or HSV-2. Interestingly, the pharmacological inhibition of the endonuclease activity of IRE-1α in HSV-1- and HSV-2-infected DCs significantly reduced apoptosis in these cells and enhanced their capacity to migrate to lymph nodes and activate virus-specific CD4^+^ and CD8^+^ T cells. These findings suggest that the activation of the IRE-1α-dependent UPR pathway in HSV-infected DCs may play a significant role in the negative effects that these viruses exert over these cells and that the modulation of this signaling pathway may be relevant for enhancing the function of DCs upon infection with HSVs.

## Introduction

Herpes simplex viruses type 1 (HSV-1) and type 2 (HSV-2) are highly prevalent in the human population worldwide (1). The global prevalence of HSV-1 and HSV-2 infection is estimated at 66.6% and 13.2%, respectively, in young adults with lifelong persistent infections and the occurrence of reactivation episodes throughout life with the possibility of viral shedding regardless of clinical symptoms (2). Herpes simplex viruses (HSVs) cause a wide range of clinical manifestations that can be mild, such as *herpes labialis*, but also severe as herpes simplex keratitis (HSK), life-threatening herpes simplex encephalitis (HSE), or neonatal encephalitis (3). Moreover, a relation between HSV infections and an increased risk of HIV infection exists (3). Because of its impact on the health of individuals, numerous strategies and approaches are constantly being evaluated to improve HSV treatment and prevent infections with these viruses (4–6). However, there are no vaccines available to date against these viruses and current therapies need improvement (4). Importantly, these viruses encode numerous molecular determinants to evade and escape the host’s innate and adaptive immune responses (7, 8). These viruses modulate early and late antiviral cellular processes, such as negative modulation of interferon production, inhibition of innate immune components, and hamper the function of adaptive immune cells (8–10).

Dendritic cells (DCs) are immune cells which play key roles in sensing, processing, and presenting virus-derived components to T cells for eliciting antiviral cytotoxic CD8^+^ T cells (CTLs) and helper T cells (CD4^+^) that support the development of B cells secreting high-affinity antiviral antibodies (11–14). Importantly, HSVs have been extensively reported to hamper DC function by interfering with the viability of these cells (12, 15), and key cellular processes related to the capacity of this cell to effectively activate T cells (8, 10, 11, 16). Given the essential roles of DCs in establishing and regulating immunity against viruses, the outcome of these cells during viral infection is critical for ultimately eliciting protective immunity (14, 17). Consistent with this notion, HSV mutants that are attenuated in DCs elicit robust protective immunity against challenges with wild-type HSV in mice (17–19).

Recently, we reported that DCs infected with HSV-2 displayed a nearly 50-fold increase in XBP1 splicing (XBP1s), compared to DCs inoculated with an attenuated mutant virus (17). Importantly, XBP1 splicing relates to the activation of the unfolded protein response (UPR) in the cell and more specifically, the inositol-requiring enzyme 1α (IRE-1α) pathway (20). The UPR is a cellular process that is activated by a significant accumulation of misfolded or unfolded proteins in the endoplasmic reticulum (ER), causing ER stress and overpassing its protein-folding capacity (21). However, the UPR may also be triggered by disruptions in homeostasis (22, 23), viral infections (24), or damage-associated inflammatory factors, such as the high mobility group box-1 protein (HMGB1) in DCs (25). Once ER stress is triggered, UPR signaling pathways can be activated by any of three routes described to date, each with its corresponding sensor: PERK (double-stranded RNA-activated protein kinase (PKR)-like ER kinase), ATF6 (activating transcription factor 6), and IRE-1α, the most conserved UPR pathway in eukaryotes (26). Importantly, UPR activation induces the transcription of factors involved in re-establishing ER homeostasis by attenuating mRNA translation, increasing the protein-folding capacity within the ER thanks to the production of protein chaperones, clearing misfolded or unfolded proteins through ER-associated protein degradation (ERAD), and by eliciting autophagy (27). However, if the cell cannot recover from ER stress, the UPR may lead to apoptosis (28).

The UPR activator IRE-1α is highly conserved among species, and once activated in the ER, it dimerizes, phosphorylates, and carries out non-traditional splicing processes in the cytoplasm over specific mRNAs (29). The most commonly studied target of IRE-1α is the mRNA of *Xbp1*, which, after removing an intron by splicing, is translated into the transcription factor XBP1s that exerts numerous UPR-related processes (30, 31). Although IRE-1α activation is frequently considered a positive process for the cell for exiting ER stress, recently, it was reported that constitutive activation of XBP1 in tumor-associated DCs inhibits the capacity of these cells to activate anti-tumor T cells (32, 33). Concomitantly, deletion of *Xbp1* in DCs promoted an anti-tumor response. These findings suggest that sustained IRE-1α activation in DCs may lead to non-optimal immune responses in the host that hamper their capacity to adequately activate T cells against tumors or other antigens (34).

Numerous viruses utilize the ER in host cells to assemble their components or direct them to other compartments, either for post-translational modifications or for reaching the cell surface. Importantly viral infection is usually accompanied by the production of significant amounts of viral proteins, and thus, infected cells are likely to undergo ER stress and UPR activation (35). Indeed, it has been described that infection with viruses such as influenza A (36), West Nile virus (37), cytomegalovirus (38), hepatitis B virus (39), and hepatitis C virus (40) activate or control at least one of the axes of the UPR response. However, because UPR activation also induces protein translation arrest and apoptosis, some viruses have evolved strategies to override or modulate ER stress and the UPR (24, 41). Notably, HSV-1 infection has been reported to interfere with PERK activation and disarm the IRE-1α-derived UPR signaling pathway early after infection in epithelial cells, as a mechanism to avoid a reduction in the production of viral proteins that are required for virion assembly (42). On the other hand, two other studies suggest that HSV-1 infection of epithelial cells activates the JNK signaling pathway, either through ERK activation or the kinase activity of IRE-1α to increase viral replication (43–45). These latter findings led us to assess the contribution of the IRE-1α pathway in DCs infected with wild-type HSV-1 or HSV-2, and its impact on the capacity of DCs to activate T cells.

We found that inhibition of the endonuclease activity of IRE-1α in HSV-1- or HSV-2-infected DCs significantly increases the viability of these cells infected with either of these two viruses, as well as their capacity to migrate to lymph nodes *in vivo* and to activate virus-specific T cells. Taken together, our findings suggest that IRE-1α activation by HSV-1 or HSV-2 in DCs leads to detrimental effects in these cells and highlights the existence of differences in IRE-1α activation and signaling events between immune and non-immune cells after HSV infection.

## Materials and Methods

### Mice

8-week-old female C57BL/6J mice were obtained from the Jackson Laboratory (Bar Harbor, ME) and maintained at the animal facility at the Pontificia Universidad Católica de Chile (Santiago, Chile). The gBT-I transgenic mouse strain, encoding an HSV-specific T cell receptor that recognizes H-2K^b^ (MHC-I)/gB_(498-505)_, was kindly shared by Dr. Francis Carbone (46) and provided by Dr. Akiko Iwasaki at Yale University, New Haven, CT, USA. The gDT-II transgenic mouse strain, encoding an HSV-specific T cell receptor that recognizes I-Ab (MHC-II)/gD_(290-302)_ was also kindly shared by Dr. Francis Carbone (47), and provided by Dr. David Taylor at University of Melbourne, Australia. Mice were handled according to the guidelines of the Institutional Ethics Committee at the Pontificia Universidad Católica de Chile and according to the approved protocols CBB-201/2013 and CEC 180821026.

### Virus Propagation

Vero cells (ATCC CCL-81) were used to propagate and titer the HSV-1 strain KOS (ATCC VR-1493), HSV-2 strain G (ATCC VR-734), HSV-1 strain K26GFP (48), and HSV-2 strain (333) ZAG (49) (kindly provided by Dr. Betsy Herold, Albert Einstein College of Medicine, New York). Briefly, T175 flasks with Vero cells monolayers were inoculated with either strain of HSV at a multiplicity of infection (MOI) 0.01 in 20 ml of Opti-MEM (Gibco, Life Technologies) and incubated at 37°C for 48 h until visible cytopathic effect. Then, the content of the flasks was pooled, and cell debris was removed twice by centrifugation at 15,000 x g for 10 min. The pellet was resuspended with 2 ml of Opti-MEM and then sonicated in an ultrasonic bath for 10 min with pulses of 15 seconds. Supernatants were stored at -80°C until use. HSV was titrated over Vero cells in flat-bottom 96 wells plates and screened for plaque production under a ZEISS Axio Vert.1A inverted microscope.

### DC Infection With HSV, Viability, Maturation and Caspase-3 Activity Assays

Dendritic cells were differentiated from hind limb bone marrow precursors of C57BL/6 mice in RPMI-1640 supplemented with 10% fetal bovine serum, 1 mM pyruvate, 2 mM Glutamine (Thermo Fisher Scientific), 1 mM non-essential amino acids, 10 mM HEPES, 100 UI/mL Penicillin/Streptomycin, and 10 ng/ml of recombinant murine granulocyte-macrophage-colony-stimulating factor (GM-CSF, GenScript), as previously described (50). The culture media was replaced every 48 h, and after 6 days of culture, DCs were then treated with 64 µM 4µ8c dissolved in dimethylformamide (DMF), 5 µM MKC-3946 dissolved in DMF or an equivalent volume of vehicle for 1 h. Afterward, DCs were infected with HSV-1 (KOS), HSV-2 (G), HSV-1 (K26GFP), or HSV-2 (333)ZAG at a MOI of 3 for 1 h at 37°C. Then, the supernatants were removed, and cells were washed with culture media and incubated again with the corresponding treatments (4µ8c, MKC-3946, or DMF). DCs were collected and analyzed for infection and viability at 12 and 24 h post-inoculation (hpi). Cell viability was assessed by flow cytometry using Zombie-NIR Fixable Viability Kit (BioLegend), over cells stained with anti-CD11c, and anti-I-A^b^ (MHC-II) antibodies (BioLegend), and/or expressing the GFP reporter protein in cells inoculated with HSV-1 K26GFP or HSV-2 (333)ZAG strain. Cells were fixed with 2% paraformaldehyde (PFA) and analyzed in a FACSCANTO II flow cytometer (BD Biosciences). To assess DC maturation, after the treatment with the IRE-1α inhibitors and HSV infection, the cells were stained with antibodies against H-2K^b^ (MHC-I), I-A^b^ (MHC-II), CD80, and CD86 (BioLegend) to evaluate their surface expression in the CD11c^+^ population by flow cytometry using a FACSVia flow cytometer (BD Biosciences). Furthermore, to evaluate the cytokine profile of the treated DCs, IL-6, IL-10, and IL-12 secretion was determined in the cell supernatants by ELISA (BioLegend) 24 h after the corresponding treatments, as previously reported (17). Recombinant murine IL-6, IL-10, and IL-12 (PeproTech) were used as standards for cytokine quantification. To evaluate the activity of caspase-3, 3x10^5^ DCs were seeded and infected with HSV-1 KOS or HSV-2 G for 1 h at MOI 3. The culture media was then replaced with fresh media. After 24 hpi, DCs were collected and centrifuged at 400 g for 5 min at 4°C. The pellet was resuspended in 100 µl of lysis buffer (HEPES 1 M, glycerol 50%, DTT 100 mM, Triton X-100 0.1%, KCl 4 M, EDTA 500 mM and protease inhibitor), incubated at 4°C for 15 min and dispensed in a 96-well black plate containing 100 µl of the Ac-DEVD-AFC (Cayman) caspase-3 substrate. Then, the mixture was incubated for 1 h and analyzed at 400 nm excitation/500 nm emission using a Cytation 5 Cell Imaging Multi-Mode Reader (BioTek). DC infection with HSV was assessed by plaque forming units (PFU) assays in triplicates, adding supernatants from infected-DCs over Vero cells monolayers seeded 24 h prior in a 96 well plate, extracting total DNA from infected cells and conducting a qPCR using 200 ng of DNA per reaction with a probe for the *U_L_30* gene. The following primers and probe were used: UL30_Fwd-GGCCAGGCGCTTGTTGGTGTA, UL30_Rev-ATCACCGACCCGGAGAGGGA, UL30_Probe-/56-FAM/CCGCCGAAC/ZEN/TGAGCAGACACCCGC/3IABkFQ/using an Applied Biosystems StepOnePlus thermocycler (51).

### DC-T Cell Antigen Presentation Assays

DCs were treated with 64 µM of 4µ8c, 5 µM of MKC-3946, or an equivalent volume of vehicle (DMF) for 1 h and then infected at an MOI of 3 with HSV-1 KOS, or HSV-2 G for 1 h (adsorption) at 37°C. Then, the supernatants were removed, washed with culture media, and incubated with the corresponding treatments. DCs were collected 6 h later and cocultured with 1x10^5^ HSV-specific T cells/well. T cells were either CD8^+^ gBT-I, or CD4^+^ gDT-II purified from spleens of gBT-I or gDT-II transgenic mice using the corresponding T cell negative-selection kits (MiltenyiBiotec). Uninfected DCs and DCs pulsed with gD_(290-302)_ or gB_(498-505)_ peptides were used as controls as previously described (17). To evaluate T cell proliferation, the cells were suspended at 10^6^/ml in PBS and incubated at 37°C for 5 min with 5 μM carboxyfluorescein succinimidyl ester (CFSE; Tocris) before they were added over DCs in cocultures. 48 h after of coculture, T cell activation and differentiation were determined by ELISA, by measuring IL-2, IL-4, IL-17 and IFN-y in the supernatants (50), and by flow cytometry using Zombie-NIR cell viability stain and antibodies against CD4, CD8, CD25, CD44, CD62L, CD69 and CD71 (BioLegend).

### DC Migration and T Cell Activation *In Vivo*

DCs were treated with 64 µM of 4µ8c, 5 µM of MKC-3946, or an equivalent volume of vehicle (DMF) for 1 h and then infected at an MOI 3 with HSV-1 KOS, or HSV-2 G for 1 h (adsorption) at 37°C. After 6 h of treatment, 5 x 10^5^ treated DCs in 50 μl were stained with 0.5 mM of CFSE and injected in the hind limb footpads of C57BL/6 mice as previously reported (52). Two days later, mice were euthanized and popliteal LNs were surgically removed and homogenized in a single cell suspension to evaluate the presence of CFSE^+^ DCs in these tissues using antibodies against CD11c and MHC-II (I-A^b^) by flow cytometry using a FACSVia flow cytometer (BD Biosciences). For assessing T cell activation *in vivo*, mice were inoculated in the hind limb with DCs as indicated above and simultaneously transferred with dye-stained virus-specific T CD4^+^ and CD8^+^ cells. Popliteal LNs were extracted and processed 48 h later to analyse T cells using the following surface markers: CD4, CD8, CD69, and CD71 (BioLegend) by flow cytometry in a BD LSRFortessa X-20 (BD Biosciences). To assess the migration of different populations of endogenous DCs from the skin into the corresponding infiltrating lymph nodes after HSV infection, mice were inoculated in the footpads with 1 x 10^6^ PFU of HSV-1 KOS, 65.3 ng of 4µ8c or 9.5 ng of MKC-3946 and 5.6 ng of CFSE in 50 µl PBS. 24 h later, popliteal LNs were extracted, processed, and analyzed by flow cytometry to determine the presence of CFSE^+^ DCs using antibodies against CD11c, MHC-II (I-A^b^), CD103 and CD207 (BioLegend).

### Statistical Analyses

Statistical analyses between experimental groups were assessed either by unpaired Student’s *t-test* (bar graphs), one-way analysis of variance (ANOVA) with Bonferroni’s multiple comparison test (three or more groups), or two-way ANOVA with Tukey’s multiple comparison test (two independent variables, with a confidence interval of 95%), as indicated, using GraphPad Prism (GraphPad Software).

## Results

### Pharmacological Inhibition of the *RNAse* Endonuclease Activity of IRE-1α in DCs Reduces HSV-Induced Cell Death

Based on our previous finding that wild-type (WT) HSV-2 triggers significant splicing of the XBP1 mRNA transcript in virus-infected DCs, as well as cell death (17), we sought to assess the contribution of IRE-1α activation of the UPR over DC viability and function during HSV-1 and HSV-2 infection.

**Figure 1:**
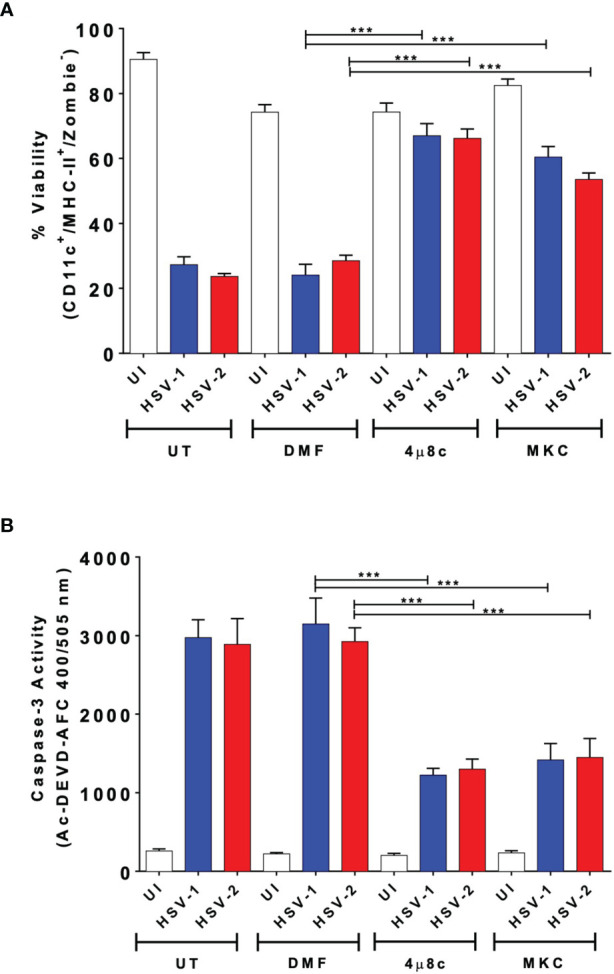
Inhibition of IRE-1α increases the viability of DCs infected with HSV-1 or HSV-2. **(A)** DCs were pre-treated with 4µ8c (64 µM) or MKC-3946 (5 µM) for 1 hour and then infected with HSV-1 KOS or HSV-2 G at an MOI 3. Cell viability was determined by flow cytometry using Zombie^®^ staining over CD11c^+^/MHC-II^+^ cells. **(B)** Caspase-3 activity in DC cultures 24 h after HSV-1 or HSV-2 infection was assessed by analyzing the fluorescence derived from the Ac-DEVD-AFC caspase-3 substrate, using a multi-mode plate reader. UT, DMF and UI correspond to untreated, vehicle-treated and uninfected DCs, respectively. Data are means ± SEM of three independent experiments. One-way ANOVA and Bonferroni’s multiple comparison test were used for statistical analyses (***p < 0.001).

First, we assessed whether inhibition of the endonuclease activity of IRE-1α impacts DC viability upon HSV infection. For this, DCs were treated either with 4µ8c or MKC-3946 (herein MKC), which inhibit the RNase endonuclease activity of IRE-1α and block the splicing of the mRNA of XBP1 (53, 54). DCs were treated with 4µ8c or MKC 1 h before infection with HSV-1 or HSV-2, while DCs treated with vehicle (DMF) were included as a control. As shown in **Figure 1A**, HSV infection of untreated DCs caused a significant reduction in the viability of these cells 24 h after viral inoculation, as determined by flow cytometry. However, when cells were treated with 4µ8c or MKC before HSV infection, DC viability was significantly higher than that observed in vehicle-treated and HSV-infected cells. Because HSV-induced DC death has been reported to be mediated by caspase-3 in murine DCs, we also measured the activation of this pro-apoptotic factor in HSV-infected DCs treated or not with 4µ8c or MKC. As observed in **Figure 1B**, 4µ8c- and MKC-treated cells displayed reduced caspase-3 activity after HSV infection in comparison to vehicle-treated and HSV-infected DCs, which is consistent with the observation that the IRE-1α inhibitors promoted increased DC viability upon HSV infection.

**Figure 2:**
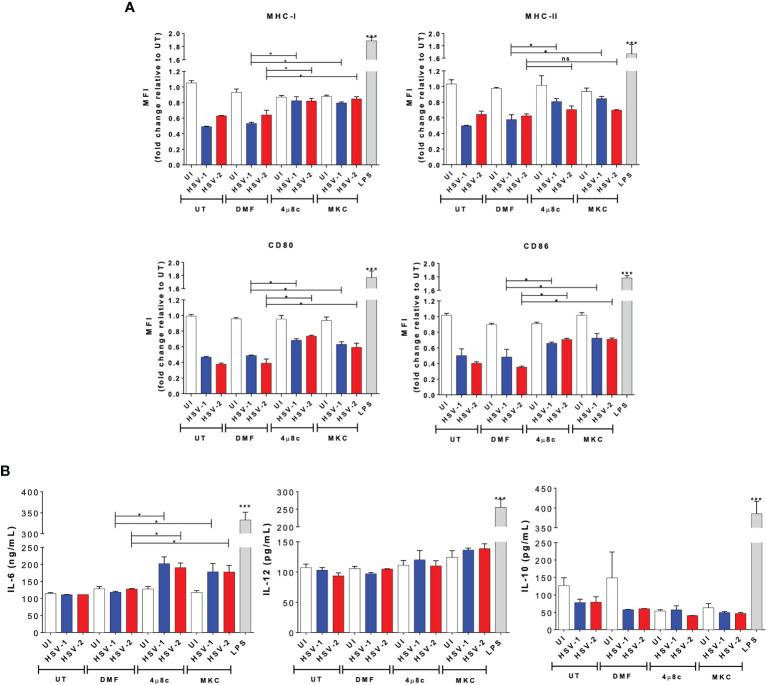
IRE-1α inhibition in HSV-infected DCs promotes cell maturation and cytokine secretion. **(A)** Expression of surface maturation markers MHC-I (H-2K^b^; upper left panel), MHC-II (I-A^b^; upper right panel), CD80 (lower left panel), and CD86 (lower right panel) determined by flow cytometry in DCs (gated on CD11c^+^/Zombie^-^ cells) at 24 h post-virus inoculation. **(B)** Supernatants from virus-inoculated DCs were assessed by ELISA to determine the presence of IL-6, IL-10 and IL-12. Data are means ± SEM of three independent experiments. Two-way ANOVA and Tukey’s multiple comparison test were used for statistical analyses (*p < 0.05, ***p < 0.001, ns, non-significant).

Secondly, to have a better understanding of the effects of blocking the endonuclease activity of IRE-1α during HSV infection in DCs, we evaluated the maturation of these cells by assessing the surface expression of costimulatory molecules, as well as the histocompatibility molecules MHC-I and MHC-II. Interestingly, we observed that DCs treated with 4µ8c or MKC and then infected with either, HSV-1 or HSV-2 displayed significantly higher levels of MHC-I, MHC-II, CD80, and CD86, except for HSV-2 infection and MHC-II, as compared to HSV-infected DCs that did not receive any drug treatment (**Figure 2A**). Furthermore, we evaluated the secretion of cytokines by these cells into the media and found that IL-6, but not IL-10 or IL-12, was significantly increased by those cells that were treated with 4µ8c or MKC and then infected with HSV (**Figure 2B**).

Overall, these results suggest that IRE-1α activation seems to play an important role in the outcome of DCs after HSV infection, with the activation of IRE-1α negatively modulating DC viability, maturation and cytokine secretion. Importantly, DC death induced by HSVs seems to be highly dependent on this pathway upon infection.

### Pharmacological Inhibition of the RNAse Endonuclease Activity of IRE-1α in DCs Inhibits the Replication of HSV-1 and HSV-2

**Figure 3:**
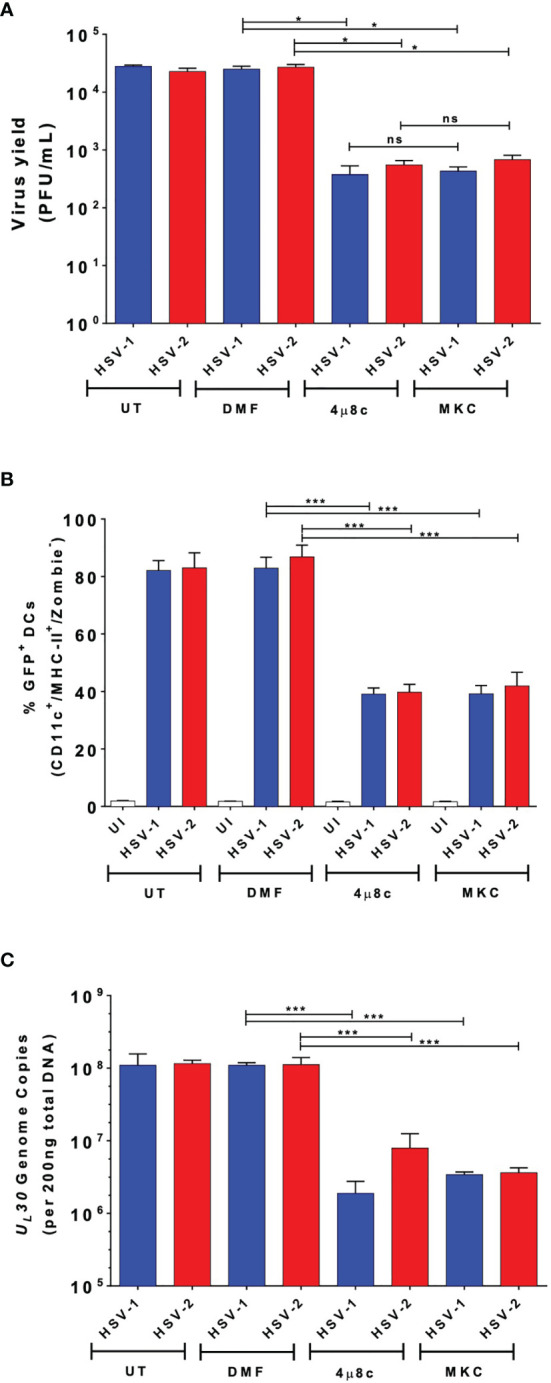
DC treatment with 4µ8c or MKC limits HSV-1 and HSV-2 yields in virus-infected DCs. **(A)** Quantification of HSV-1 KOS and HSV-2 G plaque-forming units (PFUs) recovered from the supernatants of DCs treated with 4µ8c or MKC and then infected with HSV-1 or HSV-2 24 h after infection. **(B)** Expression of the GFP-fluorescent reporter protein encoded within the genomes of HSV-1 K26GFP and HSV-2 (333)ZAG 24 h after infection of DC cultures pre-treated with 4µ8c or MKC. **(C)** Analyses of HSV-1 KOS and HSV-2 G genome copies by qPCR (*U_L_30* gene). UT, DMF and UI correspond to untreated, vehicle-treated and uninfected DCs, respectively. Data are means ± SEM of three independent experiments. One-way ANOVA and Bonferroni’s multiple comparison test were used for statistical analyses (*p < 0.05, ***p < 0.001, ns, non-significant).

To evaluate whether IRE-1α plays a role in viral replication in DCs inoculated with HSV-1 or HSV-2, we assessed whether 4µ8c or MKC treatment affects virus yields. As observed in **Figure 3A**, we observed that IRE-1α inhibition with these drugs significantly decreased the output of infectious HSV-1 and HSV-2 particles recovered from the supernatants of infected DCs. Additionally, we assessed whether DCs treated either with 4µ8c or MKC, and then infected with HSV-1 or HSV-2 viruses encoding GFP reporter genes affected GFP-derived fluorescence in these cells 24 h post-infection. As shown in **Figure 3B**, inhibition of the endonuclease activity of IRE-1α caused a significant decrease in GFP expression, as compared to HSV-infected cells that were not treated with any drug. Furthermore, 4µ8c or MKC treatment significantly decreased the amount of viral genome copies (*UL30* gene) detectable in the infected DCs at 24 h post-infection (**Figure 3C**). Therefore, these results suggest that the endonuclease activity of IRE-1α in HSV-infected DC cultures supports the replication of HSVs, along with favoring infectious particle output from these cells.

### Inhibition of the RNAse Endonuclease Activity of IRE-1α Enables HSV-Infected DCs to Activate *CD8^+^ T* Cells *In Vitro*

**Figure 4:**
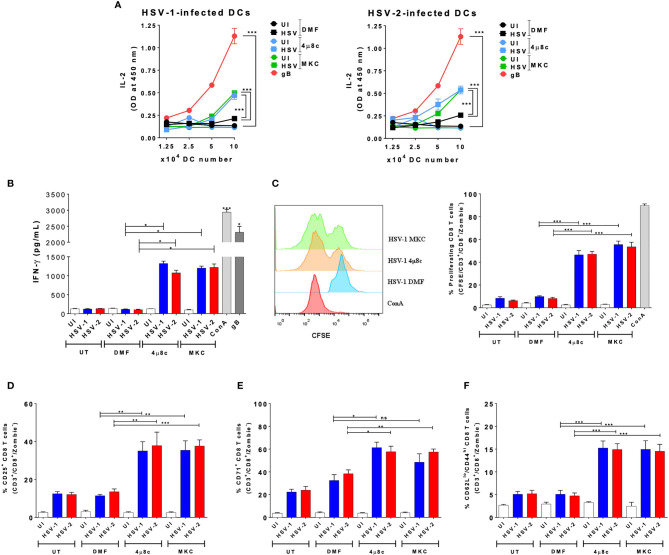
IRE-1α inhibition in HSV-infected DCs enables virus-specific CD8^+^ T cell activation. **(A)** IL-2 levels in the supernatants of cocultures of DCs infected with HSV-1 KOS (left panel) or HSV-2 G (right panel) and virus-specific gBT-I CD8^+^ T cells. **(B)** Supernatants from CD8^+^ T cells cocultured with HSV-inoculated DCs were assessed by ELISA for the presence IFN-γ. **(C)** T cell proliferation (CFSE analysis in CD3^+^/CD8^+^-gated cells) using a CFSE dilution assay 72 h after coculture with DCs treated with 4µ8c or MKC and then infected with HSV-1 KOS at an MOI 3. A representative histogram is shown in the left panel and quantification of the percentages of proliferating cells in the right panel. **(D)** Surface expression of CD25 in CD3^+^/CD8^+^ T cells cocultured with DCs treated with 4µ8c or MKC, and then infected with HSV-1 KOS or HSV-2 G. **(E)** Surface expression of CD71 in CD8^+^ T cells cocultured with DCs treated with 4µ8c or MKC, and then infected with HSV-1 KOS or HSV-2 G. **(F)** Surface expression of CD62L and CD44 in CD8^+^ T cells cocultured with DCs treated with 4µ8c or MKC, and then infected with HSV-1 KOS or HSV-2 G. UT, DMF, UI, gB and ConA correspond to untreated, vehicle-treated, uninfected DCs, HSV gB peptide-treated DCs and concavalin A (ConA), respectively. Data are means ± SEM of three independent experiments. One-way and two-way ANOVA and Tukey’s multiple comparison test were used for statistical analyses (**p < 0.01; ***p < 0.001, ns, non-significant).

Because the pharmacological inhibition of IRE-1α negatively affects the replication of HSV in DCs, while favoring cell viability, we evaluated if the inhibition of IRE-1α recovers key DC functions after HSV infection, such as their capacity to activate T cells. Therefore, we analyzed whether inhibition of IRE-1α could affect the capacity of DCs to activate virus-specific CD8^+^ T cells. To assess this possibility, we performed DC-T cell cocultures using transgenic CD8^+^ T cells that recognize epitopes derived from the HSV glycoprotein B (gB), presented in MHC-I molecules (46). Importantly, supernatants recovered from DC-T cell cocultures with HSV-infected DCs that were treated with the IRE-1α inhibitors displayed significantly more IL-2 (**Figure 4A**) and IFN-γ (**Figure 4B**) than supernatants of HSV-infected DCs alone. This finding is in line we the above-mentioned observation that DCs treated with the IRE-1α inhibitors and infected with HSV-1 display significantly more MHC-I than HSV-infected DCs that were not treated with any drug. Furthermore, we analyzed the proliferation of T cells present in the cocultures using CFSE and found that virus-specific CD8^+^ T cells displayed significantly increased proliferation when cocultured with HSV-infected DCs that were treated with any of the two inhibitors of IRE-1α (**Figure 4C**). This result is consistent with the detection of increased percentages of T cells expressing on their surface CD25, the IL-2 receptor which is associated with T cell activation (**Figure 4D**), more than T cells obtained from cocultures with DCs that were infected with HSV-1 or HSV-2 and not treated with any of the assessed drugs. Similarly, a higher percentage of CD8^+^ T cells recovered from cocultures with DCs treated with the IRE-1α inhibitors were positive for CD71, another T cell activation marker, with only HSV-1-infected treated with MKC not showing significant differences as compared to the corresponding controls (**Figure 4E**). No differences were observed between cocultures when assessing the T cell activation marker CD69 (**Supplementary Figure 1A**). Finally, to assess the acquisition of molecular markers that are associated with effector memory T cell functions that might relate to cellular functions *in vivo*, we assessed the expression of CD44 and CD62L in CD8^+^ T cells in the cocultures. Interestingly, we observed increased percentages of CD8^+^ T cells expressing CD62L^low^/CD44 ^high^ in the DC-T cell cocultures with IRE-1α inhibitor-treated and HSV-infected DCs, as compared to HSV-infected DCs alone with no drug treatment (**Figure 4F**). These results suggest a robust T cell activation. In sum, these results indicated that DC treatment with IRE-1α RNase endonuclease inhibitors before HSV infection increases their capacity to activate virus-specific CD8^+^ T cells, as compared to untreated or vehicle-treated HSV-infected DCs.

### Inhibition of IRE-1α Promotes CD4^+^ *T* Cells Activation by HSV-Infected DCs *In Vitro*

**Figure 5:**
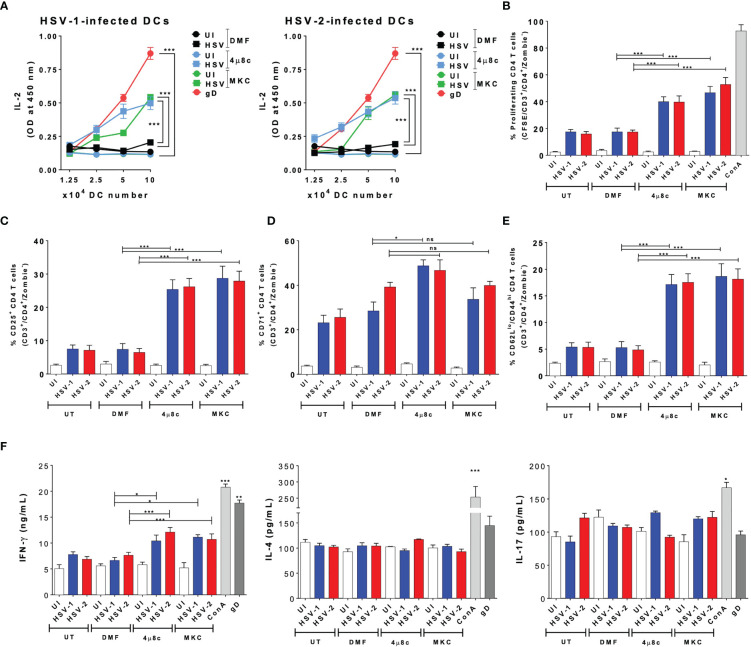
IRE-1α inhibition in HSV-infected DCs enables virus-specific CD4^+^ T cell activation. **(A)** IL-2 levels in the supernatants of cocultures with DCs infected with HSV-1 KOS (left panel) or HSV-2 G (right panel) and virus-specific gDT-II CD4^+^ T cells. **(B)** Percentages of proliferating virus-specific gDT-II CD4^+^ T cells (CFSE analysis in CD3^+^/CD4^+^-gated cells) 72 h after coculture with DCs treated with 4µ8c or MKC and then infected with HSV-1 KOS at an MOI 3. **(C)** Surface expression of CD25 in CD3^+^/CD4^+^ T cells cocultured with DCs treated with 4µ8c or MKC, and then infected with HSV-1 KOS or HSV-2 G. **(D)** Surface expression of CD71 in CD4^+^ T cells cocultured with DCs treated with 4µ8c or MKC, and then infected with HSV-1 KOS or HSV-2 G. **(E)** Surface expression of CD62L and CD44 in CD4^+^ T cells cocultured with DCs treated with 4µ8c or MKC, and then infected with HSV-1 KOS or HSV-2 G. **(F)** The supernatants obtained from CD4^+^ T cells cocultured with HSV-inoculated DCs were assessed by ELISA to determine the presence of IFN-γ, IL-4 and IL-17. UT, DMF, UI, gD and ConA correspond to untreated, vehicle treated, uninfected DCs, HSV gD peptide-treated DCs and concavalin A (ConA), respectively. Data are means ± SEM of three independent experiments. One-way and two-way ANOVA and Tukey’s multiple comparison test were used for statistical analyses (*p < 0.05, **p < 0.01, ***p < 0.001, ns, non-significant).

Subsequently, we proceeded to evaluate the role of IRE-1α on the HSV-1- and HSV-2-infected DCs to activate virus-specific CD4^+^ T cells. Similar to the DC-CD8^+^ T cell cocultures, we performed DC-T cell cocultures using transgenic virus-specific CD4^+^ T cells that recognize a specific epitope of the HSV glycoprotein D (gD) presented in MHC-II molecules (55). Importantly, we found that in cocultures with DCs treated with either of the IRE-1α inhibitors and infected with HSV-1 or HSV-2, significantly more CD4^+^ T cell activation was observed, as compared to cocultures with untreated and HSV-infected DCs. Indeed, the supernatants obtained from DC-T cell cocultures with DCs treated with 4µ8c or MKC and then infected with HSV displayed significantly more IL-2 than those from cocultures with vehicle-treated DCs and HSV infection (**Figure 5A**). In addition, similar to the results reported for the CD8^+^ T cells, we found significantly higher proliferation levels of virus-specific CD4^+^ T cells in the cocultures with DCs treated with inhibitors for IRE-1α and infected with HSV, as compared to HSV-infected DCs without any drug, which showed lower levels of proliferation (**Figure 5B**). Moreover, the percentages of virus-specific CD25^+^ T cells were higher in the cocultures with the IRE-1α inhibitors, as compared to T cells obtained from cocultures with HSV-1 or HSV-2 infected DCs without any drug, as measured by flow cytometry (**Figure 5C**). We further observed increased percentages of virus-specific CD4^+^ T cells positive for CD71 in the cocultures of 4µ8c- or MKC-treated and HSV-infected DCs, as compared to cocultures without these inhibitors (**Figure 5D**). For CD69^+^ expression in CD4^+^ T cells, we only observed significant differences in the percentages of CD69^+^ CD4^+^ T cells in the cocultures with DCs treated with 4µ8c and infected with HSV-1, as compared to the other coculture conditions (**Supplementary Figure 1A**). Similar to the results with the CD8^+^ virus-specific T cells described above, increased percentages of CD4^+^ T cells expressing CD62L^low^/CD44 ^high^ we observed in the DC-T cell cocultures with the IRE-1α inhibitor-treated and HSV-infected DCs, as compared to HSV-infected DCs with no drug treatment (**Figure 5E**). Finally, we evaluated the secretion of the cytokines IFN-γ, IL-4 and IL-17 in these cocultures to determine the polarization phenotype of the activated CD4^+^ T cells. Overall, we found significantly increased levels of IFN-γ secretion in the T cells cocultured with DCs treated with 4µ8c or MKC and then HSV-infected, as compared to cocultures with HSV-1 or HSV-2 infected DCs only (**Figure 5F**).

In summary, these results indicate that inhibition of the endonuclease activity of IRE-1α previous to HSV infection largely recovers the capacity of DCs to activate virus-specific CD4^+^ T cells.

### Inhibition of IRE-1α in HSV-Infected DCs Enhances Their Migration Capacity and Promotes Virus-Specific T Cell Activation *In Vivo*

**Figure 6:**
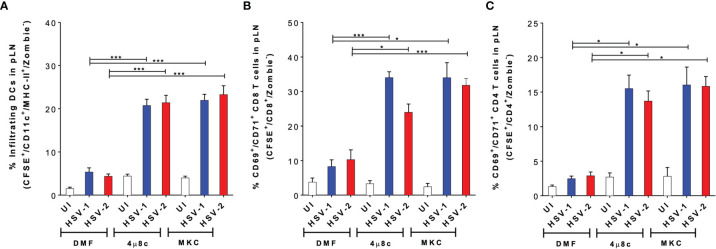
Inhibition of IRE-1α in HSV-infected DCs promotes the migration of DCs from the skin to draining lymph nodes and the activation of virus-specific T cells *in vivo*. **(A)** Migration of DCs treated with 4µ8c or MKC and then inoculated with HSV-1 or HSV-2, from the hindlimb footpads to draining popliteal lymph nodes (pLNs). Inoculated DCs were stained with CFSE and detected in the draining lymph node (CFSE^+^-gated, then CD11c^+^/MHC-II^+^/Zombie^–^-cells-analysed). **(B)** CD69 and CD71 surface expression in virus-specific gBT-I CD8^+^ T cells in pLNs 48 h after the inoculation of IRE-1α inhibitor-treated and HSV-1 or HSV-2-infected DCs in the footpads. **(C)** CD69 and CD71 surface expression in virus-specific gDT-II CD4^+^ T cells 48 h after the inoculation of IRE-1α inhibitor-treated and HSV-1 or HSV-2-infected DCs in the footpads. One-way ANOVA with Tukey’s multiple comparison test were used for statistical analyses (*p < 0.05, ***p < 0.001). Data are means ± SEM (n = 2 mice/group).

To gain a better understanding of the results described above, *in vivo* experiments were performed to assess the migration capacity of the treated DCs using a previously reported DC migration assay (17, 52). DCs treated with 4µ8c or MKC and then infected with HSV-1- or HSV-2- were stained with CFSE and inoculated into the hind limb footpads of mice. Then, the popliteal lymph nodes (pLNs) were recovered 48 h later to assess the migration of the CFSE^+^ DCs to this tissue. As shown in **Figure 6A**, we observed a significantly higher proportion of CFSE^+^ DCs in the pLNs when these cells were treated with 4µ8c or MKC and then infected with HSV, as compared to HSV-infected DCs alone. Furthermore, to assess the capacity of the transferred DCs to activate T cells *in vivo*, we measured the percentages of CD69^+^ and CD71^+^ virus-specific CD8^+^ and CD4^+^ T cells, previously transferred into the mice, in the pLNs 48 h after inoculation of the DCs. In all cases, we observed significantly higher percentages of CD69^+^/CD71^+^/CD8^+^ (**Figure 6B**) and CD69^+^/CD71^+^/CD4^+^ (**Figure 6C**) T cells in mice inoculated with the DCs treated with the IRE-1α inhibitors and HSV-infected, as compared to those inoculated with the HSV-infected DCs that were not pharmacologically treated. Additionally, we performed an *in vivo* assay to determine the migration of local DCs, residing in the skin of the mice, to the corresponding LNs when injecting the IRE-1α inhibitors at the site of HSV-1 inoculation in the hindlimb footpads. Interestingly, in this case we found that the injection of the inhibitors of IRE-1α in the footpads, in which HSV-1 was also inoculated together with the dye CFSE to track migrating DCs, promoted the migration of DCs (CD11c^+^/MHC-II^+^) into the pLNs (**Supplementary Figure 2A**), particularly dermal DCs (CD11c^+^/MHC-II^+^/CD103^+^/CD207^+^) (**Supplementary Figure 2B**) and Langerhans cells (CD11c^+^/MHC-II^+^/CD103^-^/CD207^+^) from the periphery (**Supplementary Figure 2C**).

Altogether, these results suggest that the inhibition of the endonuclease activity of IRE-1α in HSV-infected DCs, or at the site of inoculation of HSV-1 recovers the migrating capacity of DCs to the draining LNs and promotes the stimulation of virus-specific T cells *in vivo.*

## Discussion

Herpes simplex viruses have evolved numerous molecular factors to evade the host immune response and alter the optimal function of immune cells. Notably, DCs are a key component in the initiation and regulation of the immune response against viruses, and HSVs target these cells to interfere with their functions. HSV-1 and HSV-2 hamper numerous cellular processes in DCs and ultimately promote their death (12, 15), which may result in the inability of the host to establish an effective and robust antiviral response against these viruses (17). Because of the crucial role of DCs in establishing a protective adaptive immunity against HSVs, it is important to understand the mechanisms by which these viruses modulate DC function and to identify novel strategies for promoting their immune-activating functions against these viruses (12, 16, 17, 56, 57).

Some reports suggest that HSV-1 infection disarms the UPR response. Indeed, this was described for epithelial cells infected with HSV-1, in which only the ATF6 signaling pathway was activated early during infection, yet without the concomitant expression of target chaperones (42). A later study reported that HSV-1 suppresses the IRE-1α signaling pathway in epithelial cells thanks to the virion host shutoff protein (vhs, encoded by the *UL41* gene) by reducing XBP1 mRNA levels during ER-induced stress (58). Another study further analyzed the roles of the RNase and kinase activities of IRE-1α in the replication of HSV-1 in endometrium epithelial cells, and found that activating the RNase activity of IRE-1α, or inhibiting the kinase activity of IRE-1α led to reduced viral replication (59). The latter is consistent with a recent study that reported that inhibition of the RNase activity of IRE-1α reduces HSV replication within infected cells. However, the observation was particularly mediated by abolishing an anti-apoptotic effect in HSV-infected cells (58). In contrast, a more recent study found that treating the epithelial cell lines HeLa or HEC-1 cells with an ER stress inhibitor, namely tauroursodeoxycholic acid (TUDCA), increased eIF2α phosphorylation, which induced the phosphorylation and initial activation of PERK, ATF6, and the ER sensor chaperone protein BiP. Consequently, BiP would bind to the luminal domains of PERK or IRE-1α to inhibit their activation during HSV-1 infection (60).

Although numerous reports suggest that HSV negatively modulates the UPR response in infected epithelial cells, we reported in a previous study that this may not be the case in DCs (17). This notion is reinforced by the findings described herein, which indicate opposing effects between DCs and epithelial cells infected with HSV, as inhibiting the RNase activity of IRE-1α with 4µ8c or MKC significantly reduced virus yields in the former cells. Furthermore, these treatments led to increased viability of DCs after infection, suggesting that the activation of the IRE-1α pathway during HSV infection likely directs the cell fate towards apoptosis. This effect is opposing to that seen in other cells, such as murine embryonic fibroblasts (MEFs), in which the inhibition of UPR is understood as a viral mechanism to promote viral replication, by conferring the cells increased resistance to apoptosis and hence, increased cell survival serving as a substrate for the virus (45).

However, the activation of the UPR response can also lead to apoptosis (30, 44), and the findings described herein are somewhat in line with effects reported for other viruses on this cellular process, such as the influenza A virus (36), and the Japanese encephalitis virus (61), in which cases it was observed that inhibition of XBP1 mRNA splicing was associated to decreased viral replication, although those findings were described in neuroblastoma cell lines. Overall, the differences we observe in this study regarding the activation of the UPR are likely dependent on the cell type, which adds on to other differences observed for HSVs when comparing immune and non-immune cell responses to these viruses. For instance, upon infection HSVs elicit anti-apoptotic effects in epithelial cells and neurons, but apoptotic outcomes in DCs (12, 15, 16) and T cells (8). Interestingly, our findings suggest that IRE-1α-mediated activation of the UPR response by HSVs in DCs is a mechanism evolved by these viruses to hamper the T cell-activating functions of these cells and, furthermore, elicit DC death. Previous reports have evidenced that HSV impairs the capacity of DCs to activate T cells and that this effect is likely mediated through the apoptosis of both, DCs and T cells in cocultures, or by the negative modulation of TCR signaling in T cells (62–64). Although we observed some levels of caspase-3 activity herein in the DCs treated with the IRE-1α inhibitors, despite increased viability in these cells in these conditions, it is possible that these levels might be below the threshold for inducing apoptosis, while these results could also account for other viral factors activating this pathway or by unspecific effects elicited by these IRE-1α inhibitors (65, 66).

On the other hand, the results described herein suggest that HSVs induce the IRE-1α pathway in DCs to reduce antigen presentation to virus-specific CD4^+^ or CD8^+^ T cells, supported by the differences observed for MHC-I and MHC-II surface expression in treated and infected-DCs. Importantly, these results are consistent with a recent report associating decreased MHC-I expression with IRE-1α activation in DCs (33). However, based on our current results we cannot provide a molecular mechanism relating the increased MHC expression observed as a consequence of DC treatment with the inhibitors of the endonuclease activity of IRE-1α. Interestingly, a recent study reported that DCs inoculated with an HSV-1 mutant with the gene encoding γ34.5 deleted significantly improved DC outcome and elicited protective antiviral immunity against viral challenge (67). Noteworthy, the γ34.5 factor negatively modulates the PERK UPR response through the inhibition of eIF2α phosphorylation, while promoting DC maturation through TBK1 activation (68, 69). Nevertheless, it will be interesting to determine if particular viral factors expressed in DCs versus epithelial cells may play particular roles in activating the IRE-1α pathway in DCs.

Importantly, previous reports indicate that T cell activation *in vivo* during HSV infection requires that dermal DCs relay HSV antigens to the lymph nodes by capturing HSV-infected apoptotic Langerhans cells in the skin (70–72). Interestingly, in this study we found that DCs treated with the IRE-1α inhibitors and infected with HSV, that were inoculated in the hindlimb footpads of mice, displayed an enhanced capacity to migrate to the draining LNs when compared to HSV-infected DCs that were not treated with any drugs. This finding indicates that the inhibition of IRE-1α in DCs infected with these viruses restores an important function in these cells, which is generally associated with antigen presentation to T cells in the LNs. Indeed, this enhanced migration capacity of DCs was accompanied with increased virus-specific T cell activation in this tissue, suggesting that an anti-HSV response in these animals was likely initiated by these DCs. However, whether the T cell response induced by these DCs may be sufficient to confer protection against a later challenge with HSV remains to be determined in future experiments. Furthermore, we found that the injection of the inhibitors of the endonuclease activity of IRE-1α, together with HSV-1 in the skin promoted both, Langerhans cell and dermal DC migration from the periphery to draining LNs after HSV-1 infection, suggesting that the presence of these drugs, at the site of infection with HSV-1, affects the capacity of Langerhans cells to migrate the LNs, allowing them to reach this tissue together with dermal DCs and thus, IRE-1α inhibition may help bypass to some extent the relay indicated above and favor HSV antigen presentation to T cells. Nevertheless, whether the increased DC migration that was observed in this assay is due to a direct inhibition of IRE-1α in HSV-infected DCs, IRE-1α inhibition in other cell types adjacent to the migrating DCs, or to effects of IRE-1α inhibition on tissue inflammation in the skin upon HSV-1 infection remains to be assessed experimentally.

Taken together, our results suggest that the IRE-1α pathway likely plays an important role in the detrimental effects exerted by HSV-1 and HSV-2 over DC function and consequently virus-specific T cell activation. Altogether, our findings propose that the IRE-1α pathway may be a relevant target for new antiviral strategies aiming at improving the immune response against HSVs.

## Data Availability Statement

The original contributions presented in the study are included in the article/**Supplementary Material**. Further inquiries can be directed to the corresponding author.

## Ethics Statement

The animal study was reviewed and approved by Institutional Ethics Committee at the Pontificia Universidad Católica de Chile, protocols CBB-201/2013, CEC 180504003 and CEC 180821026.

## Author Contributions

ET, AR-D, MF, and PG designed experiments. ET, AR-D, MF, LD, TP-P, and FI conducted experiments. ET, AR-D, PG, CR, SB, and AK analyzed the data and wrote the manuscript. All authors contributed to the article and approved the submitted version.

## Funding

This work was funded by the Agencia Nacional de Investigación y Desarrollo (ANID) - Millennium Science Initiative Program - ICN09_016: Millennium Institute on Immunology and Immunotherapy (ICN09_016; former P09/016-F), FONDECYT grant #1190864 and supported by FONDEQUIP EQM130158 from ANID and the Regional Government of Antofagasta through the Innovation Fund for Competitiveness FIC-R 2017 (BIP Code: 30488811-0). MF and ET are ANID fellows #21191390 and #21211300, respectively.

## Conflict of Interest

The authors declare that the research was conducted in the absence of any commercial or financial relationships that could be construed as a potential conflict of interest.

## Publisher’s Note

All claims expressed in this article are solely those of the authors and do not necessarily represent those of their affiliated organizations, or those of the publisher, the editors and the reviewers. Any product that may be evaluated in this article, or claim that may be made by its manufacturer, is not guaranteed or endorsed by the publisher.

## Supplementary Material

Supplementary Figure 1 CD69 and CD71 expression on the surface of CD8^+^ and CD4^+^ T cells in cocultures with IRE-1α inhibitor-treated and HSV-infected DCs. A. Surface expression of CD69 in CD3^+^/CD8^+^ T cells (left panel), or CD3^+^/CD4^+^ T cells (right panel) cocultured with DCs treated with 4μ8c or MKC, and then infected with HSV-1 KOS or HSV-2 G. B. Mean fluorescence intensity (MFI) of CD69 staining in CD3^+^/CD8^+^ T cells (left panel), or CD3^+^/CD4^+^ T cells (right panel) cocultured with DCs treated with 4μ8c or MKC, and then infected with HSV-1 KOS or HSV-2 G. C. MFI of CD71 staining in CD3^+^/CD8^+^ T cells (left panel), or CD3^+^/CD4^+^ T cells (right panel) cocultured with DCs treated with 4μ8c or MKC, and then infected with HSV-1 KOS or HSV-2 G. UT, DMF, UI and gB or gD correspond to untreated, vehicle-treated, uninfected DCs, and gB- or gD-peptide treated DCs, respectively. Data are means ± SEM of three independent experiments. One-way and two-way ANOVA and Tukey’s multiple comparison test were used for statistical analyses (*p < 0.05, ***p < 0.001, ns, non-significant).

Supplementary Figure 2
*In vivo* migration of local skin resident DC populations into popliteal LNs after footpad injection with IRE-1α endonuclease inhibitors and HSV-1 inoculation. A. Detection of CD11c^+^/MHC-II^+^ migrating DCs from the hindlimb footpads to pLNs 24 h after injection of HSV-1, 4μ8c or MKC inhibitors and CFSE tracking dye (CFSE^+^-gated cells analysed for CD11c^+^/MHC-II^+^/Zombie^-^). B. Detection of CD103^+^/CD207^+^ migrating dermal DCs from the hindlimb footpads to pLNs 24 h after injection of HSV-1, 4μ8c or MKC inhibitors and CFSE tracking dye (CFSE^+^-gated cells, analysed for CD11c^+^/MHC-II^+^/CD103^+^/CD207^+^/Zombie^-^). C. Detection of CD103^-^/CD207^+^ migrating Langerhans cells from the hindlimb footpads to pLNs 24 h after injection of HSV-1, 4μ8c or MKC inhibitors and CFSE tracking dye (CFSE^+^-gated cells analysed for CD11c^+^/MHC-II^+^/CD103^-^/CD207^+^/Zombie^-^). One-way ANOVA with Tukey’s multiple comparison test were used for statistical analyses (*p < 0.05, ***p < 0.001, ns, non-significant). Data are means ± SEM (n = 2 mice/group).
