# Supplementary figures and images for: Pharmacological Inhibition of IRE-1 Alpha Activity in Herpes Simplex Virus Type 1 and Type 2-Infected Dendritic Cells Enhances T Cell Activation
Source: Front Immunol. 2022 Jan 5;12:764861. doi: 10.3389/fimmu.2021.764861 (PMC8766714; doi:10.3389/fimmu.2021.764861)

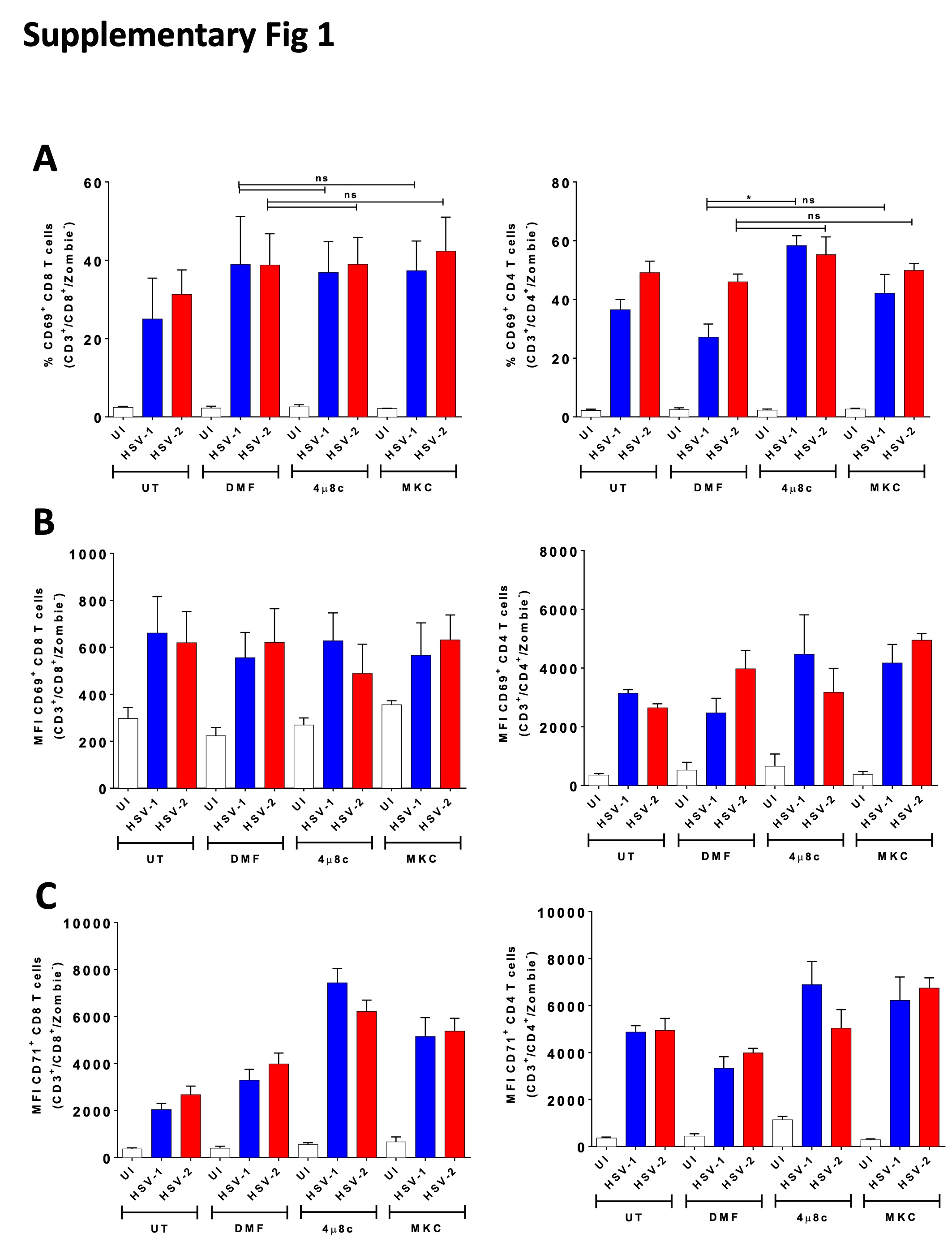

Supplement: Supplementary Figure 1 — CD69 and CD71 expression on the surface of CD8+ and CD4+ T cells in cocultures with IRE-1α inhibitor-treated and HSV-infected DCs. A. Surface expression of CD69 in CD3+/CD8+ T cells (left panel), or CD3+/CD4+ T cells (right panel) cocultured with DCs treated with 4μ8c or MKC, and then infected with HSV-1 KOS or HSV-2 G. B. Mean fluorescence intensity (MFI) of CD69 staining in CD3+/CD8+ T cells (left panel), or CD3+/CD4+ T cells (right panel) cocultured with DCs treated with 4μ8c or MKC, and then infected with HSV-1 KOS or HSV-2 G. C. MFI of CD71 staining in CD3+/CD8+ T cells (left panel), or CD3+/CD4+ T cells (right panel) cocultured with DCs treated with 4μ8c or MKC, and then infected with HSV-1 KOS or HSV-2 G. UT, DMF, UI and gB or gD correspond to untreated, vehicle-treated, uninfected DCs, and gB- or gD-peptide treated DCs, respectively. Data are means ± SEM of three independent experiments. One-way and two-way ANOVA and Tukey’s multiple comparison test were used for statistical analyses (*p < 0.05, ***p < 0.001, ns, non-significant). [file Image_1.jpeg]

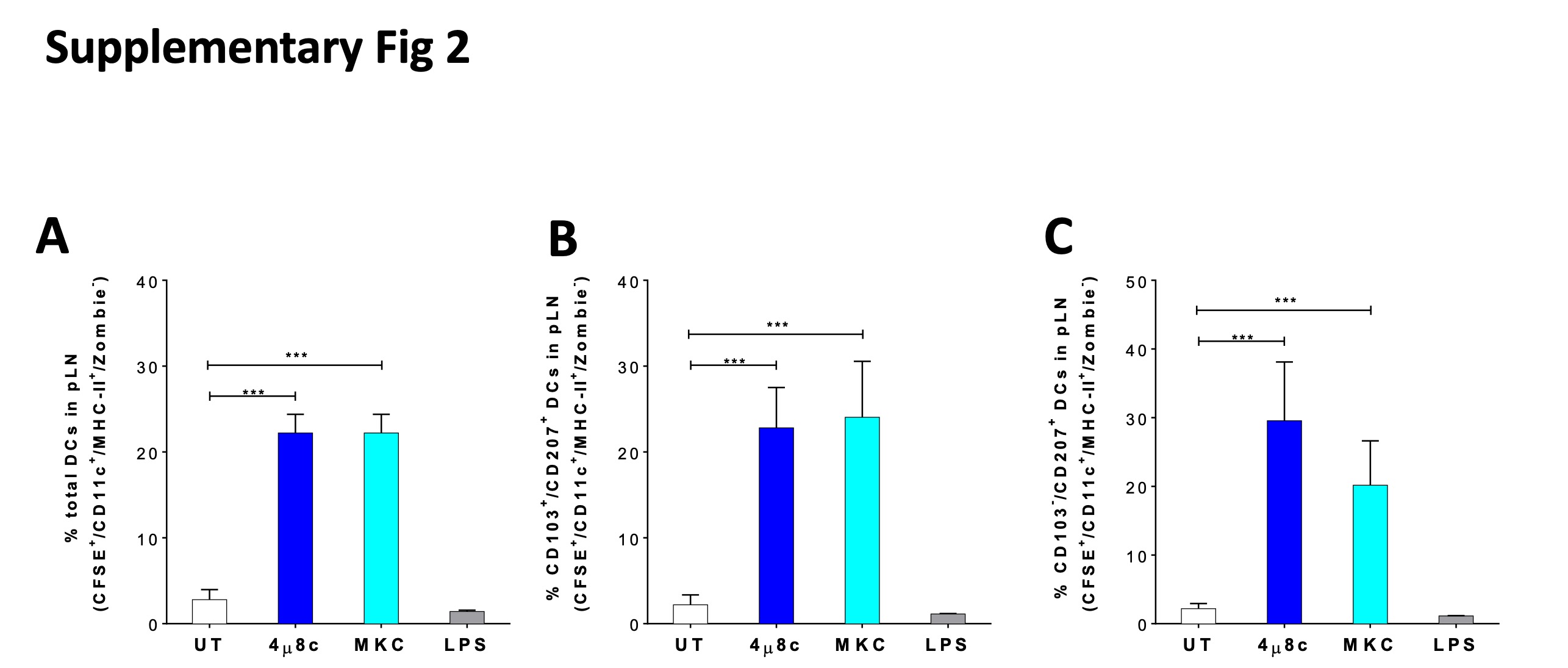

Supplement: Supplementary Figure 2 — In vivo migration of local skin resident DC populations into popliteal LNs after footpad injection with IRE-1α endonuclease inhibitors and HSV-1 inoculation. A. Detection of CD11c+/MHC-II+ migrating DCs from the hindlimb footpads to pLNs 24 h after injection of HSV-1, 4μ8c or MKC inhibitors and CFSE tracking dye (CFSE+-gated cells analysed for CD11c+/MHC-II+/Zombie-). B. Detection of CD103+/CD207+ migrating dermal DCs from the hindlimb footpads to pLNs 24 h after injection of HSV-1, 4μ8c or MKC inhibitors and CFSE tracking dye (CFSE+-gated cells, analysed for CD11c+/MHC-II+/CD103+/CD207+/Zombie-). C. Detection of CD103-/CD207+ migrating Langerhans cells from the hindlimb footpads to pLNs 24 h after injection of HSV-1, 4μ8c or MKC inhibitors and CFSE tracking dye (CFSE+-gated cells analysed for CD11c+/MHC-II+/CD103-/CD207+/Zombie-). One-way ANOVA with Tukey’s multiple comparison test were used for statistical analyses (*p < 0.05, ***p < 0.001, ns, non-significant). Data are means ± SEM (n = 2 mice/group). [file Image_2.jpeg]
